# Mitochondrial protein import - Functional analysis of the highly diverged Tom22 orthologue of *Trypanosoma brucei*

**DOI:** 10.1038/srep40738

**Published:** 2017-01-17

**Authors:** Jan Mani, Samuel Rout, Silvia Desy, André Schneider

**Affiliations:** 1 Department of Chemistry and Biochemistry, University of Bern, Freiestrasse 3, CH-3012 Bern, Switzerland

## Abstract

The β-barrel protein Tom40 and the α-helically anchored membrane protein Tom22 are the only universally conserved subunits of the protein translocase of the mitochondrial outer membrane (TOM). Tom22 has an N-terminal cytosolic and a C-terminal intermembrane space domain. It occurs in two variants: one typified by the yeast protein which has a cytosolic domain containing a cluster of acidic residues, and a shorter variant typified by the plant protein that lacks this domain. Yeast-type Tom22 functions as a secondary protein import receptor and is also required for the stability of the TOM complex. Much less is known about the more widespread short variant of Tom22, which is also found in the parasitic protozoan *Trypanosoma brucei*. Here we show that the intermembrane space domain of trypanosomal Tom22 binds mitochondrial precursor proteins and that it is essential for normal growth and mitochondrial protein import. Moreover, complementation experiments indicate that the intermembrane space domain cannot be replaced by the corresponding regions of the yeast or plant Tom22 orthologues. Lack or replacement of the short cytosolic domain, however, does not interfere with protein function. Finally, we show that only the membrane-spanning domain of trypanosomal Tom22 is essential for assembly of the trypanosomal TOM complex analogue.

The evolution of the mitochondrial protein import system was a key step that transformed the endosymbiotic ancestor of the mitochondrion into a true organelle that is largely under the control of the nucleus^1^,^2^. Protein import across the mitochondrial outer membrane (OM) is mediated by the translocase of the mitochondrial OM (TOM) which has been analyzed in great detail in the yeast *Saccharomyces cerevisiae*. The yeast TOM complex consists of the 7 subunits: Tom40, Tom22, Tom5, Tom6, Tom7 and the primary receptors Tom20 and Tom70^3^. Bioinformatic analysis and biochemical characterization of TOM in plants and trypanosomes revealed that only Tom40 and Tom22 are conserved in all eukaryotes^2^. Tom40 is a β-barrel membrane protein of the voltage-dependent anion channel (VDAC)-like protein family that forms the pore across which proteins are imported into mitochondria^4^. Tom22 is an α-helically anchored membrane protein that is tightly associated with Tom40 and that has soluble N- and C-terminal domains exposed to the cytosol and the intermembrane space (IMS), respectively^5^,^6^,^7^,^8^. Yeast Tom22 has a molecular weight of 18 kDa and functions as a secondary receptor. It interacts with the primary receptors Tom20 and Tom70. The N-terminal cytosolic domain of yeast Tom22 contains a cluster of acidic amino acids and also the C-terminal IMS domain is enriched for acidic residues^7^,^8^. Both regions have been implicated in presequence binding, although the acidic residues of the cytosolic domain can be replaced without affecting the interaction with presequences^9^. Moreover, independent of its receptor function yeast Tom22 is required for TOM assembly into a 450 kDa complex^10^.

An orthologue of Tom22, termed Tom9, has been identified in isolated TOM of higher plants^11^,^12^. It has a molecular weight of 9 kDa and has a short cytosolic domain that essentially lacks acidic residues. The amino acid composition of its IMS domain however is similar to yeast Tom22 and is able to functionally replace the corresponding region of the yeast protein^13^. It is not known whether plant Tom9 is essential and whether, as its yeast counterpart, it mediates TOM complex stability.

Recently, the functional analogue of the TOM complex has been characterized in the parasitic protozoa *Trypanosoma brucei.* Trypanosomes belong to the eukaryotic supergroup of the excavates which arguably diverged very early from essentially all other eukaryotes^14^,^15^. The trypanosomal TOM was therefore termed archaic TOM (ATOM). It consists of 6 subunits: ATOM40, ATOM14, ATOM12, ATOM11 and the primary receptors ATOM46 and ATOM69^16^,^17^. ATOM14 is a 14 kD protein whose transmembrane domain and flanking regions show similarity to Tom22 and Tom9 when analyzed by HHPred^18^ (Fig. 1). However it lacks the conserved proline residue in the transmembrane domain. Similar to plant Tom9 the cytosolic domain of ATOM14 lacks acidic amino acids and is even shorter than in the plant protein. In contrast, ATOM14 has an IMS domain that is longer than the one in the yeast or plant Tom22 orthologues. ATOM14 is essential for normal growth in both procyclic and bloodstream form cell lines including in an engineered bloodstream form cell line that can grow in the absence of kDNA. Moreover as Tom22 in yeast, it plays an important role in (A)TOM assembly, since in its absence much less of the ATOM complex is formed^16^.

Yeast-type Tom22 proteins that have a cytosolic cluster of acidic amino acids are restricted to the eukaryotic supergroup of the Opisthokonts which includes fungi and metazoans^19^. Tom22 orthologues in all other supergroups lack the acidic cluster and therefore are of the plant-type^2^,^13^.

Here we present an experimental analysis of trypanosomal ATOM14, a remote orthologue of Tom22, which as the plant-type Tom22 lacks an acidic cluster in its cytosolic domain. We have investigated the contribution of the cytosolic, membrane-spanning and IMS domains of ATOM14 to the specific functions of the protein. Moreover, we have analyzed to which extent domains of the yeast and the plant Tom22 orthologues can function in the context of the trypanosomal protein. All these expriments have been done using insect-stage *T. brucei.*

## Results

### ATOM14 is an ATOM complex subunit with a cytosol-facing N-terminus

**Figure 1:**
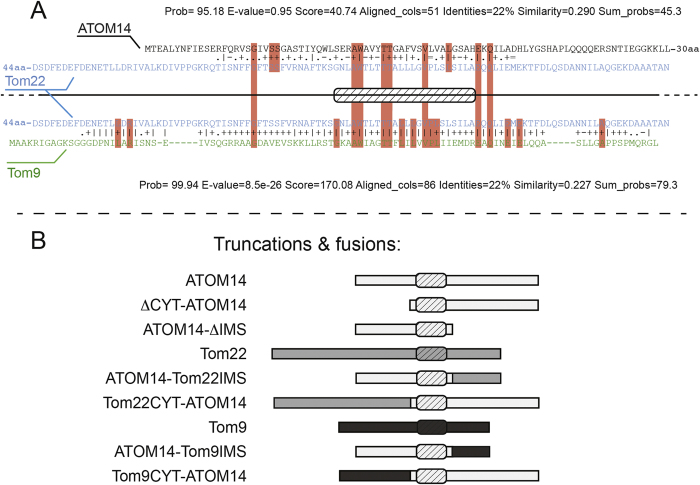
Sequence similarities between ATOM14/Tom22 and Tom22/Tom9 and predicted domain structures of the proteins. (**A**) Top, alignment of Tom22 of *S. cerevisiae* (blue) with ATOM14 of *T. brucei* (black). Bottom, alignment of Tom22 (blue) with Tom9 of *A. thaliana* (green). Both alignments were produced using HHPred. Identical residues in the Tom22-ATOM14 or the Tom22-Tom9 alignments are indicated by red boxes. Identical residues present in all three proteins (Tom22/ATOM14/Tom9) are shown in the large red boxes. The transmembrane segment predicted by the HMMTOP server (http://www.enzim.hu/hmmtop/) is indicated by the cross-hatched box. (**B**) Cartoon of all ATOM14 (grey bar), Tom22 (white bar) and Tom9 (black bar) (fusion)-proteins and their designations used in this study. Crosshatched box: predicted transmembrane domain.

Besides ATOM40 which likely is a highly diverged member of the VDAC-like protein family, ATOM14 is the only ATOM complex subunit that shows any similarity to components of the TOM complexes of yeast and plants. HHPred^18^ reveals that this similarity is restricted to an approximately 50 amino acid long segment (amino acids 12–63) which includes the predicted transmembrane domain (amino acids 33–56) (Fig. 1A). This suggests that, despite their different molecular weights ATOM14, Tom22 and Tom9 share a common ancestor. Thus, with the aim to perform a comparative functional analysis of the different Tom22 orthologues, we investigated trypanosomal ATOM14 in more detail.

**Figure 2:**
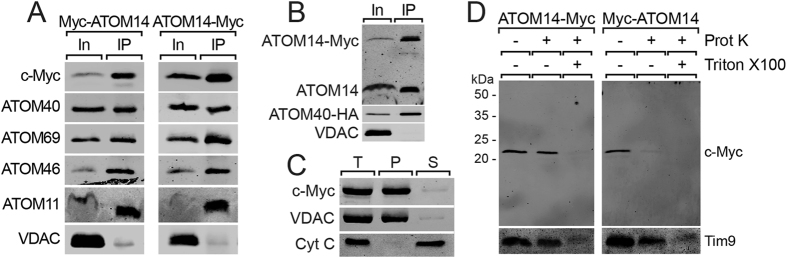
ATOM14 is an ATOM complex subunit with a cytosol-facing N-terminus. (**A**) Digitonin-extracted crude mitochondrial fractions of procyclic *T. brucei* cell lines expressing N- or C-terminally c-Myc-tagged ATOM14, respectively, were subjected to co-immunprecipitations using an anti-c-Myc antiserum. Immunoblots containing 5% input (In) and 100% eluate (IP) were probed for the presence of the tagged proteins and the indicated ATOM complex subunits. VDAC serves as a control. (**B**) C-terminally c-Myc-tagged ATOM14 was immunoprecipitated using crude mitochondrial fractions from a cell line co-expressing C-terminally c-Myc-tagged ATOM14 and C-terminally HA-tagged ATOM40. Immunoblots containing 5% input (In) and 100% eluate (IP) were probed with an anti-ATOM14 antiserum that recognizes both the tagged als well as the endogenous ATOM14. HA-tagged ATOM40 and VDAC serve as a controls. (**C**) Immunoblots of the total (T), pellet (P) and supernatant (S) fractions of carbonate extracted mitochondria isolated from cells expressing C-terminally c-Myc-tagged ATOM14 were analyzed by anti-c-Myc antiserum. VDAC and cytochrome C (Cyt C) serve as marker for an integral and peripheral membrane protein, respectively. (**D**) Immunoblots of a protease protection assay using gradient-purified mitochondria isolated from cell lines expressing N- or C-terminally c-Myc-tagged ATOM14, respectively, analyzed by anti-c-Myc antiserum. The IMS protein Tim9 serves as a control.

ATOM14 was initially discovered as a mitochondrial protein that co-immunoprecipitates with ATOM40^16^. Figure 2A shows that in line with this finding immunoprecipitations of either N- or C-terminally c-Myc-tagged ATOM14 recovers all other ATOM subunits but not the highly abundant OM protein VDAC that serves as a control. Since we do not have an antiserum against ATOM12, its presence in the eluates could not be confirmed. Interestingly, not only tagged ATOM14 but also the untagged version of the protein was recovered in the eluate indicating that the ATOM complex contains more than one molecule of ATOM14 (Fig. 2B). Moreover, carbonate extraction at high pH shows that ATOM14 is recovered in the pellet fraction supporting the prediction that it is an integral membrane protein (Fig. 2C).

We isolated mitochondria from cell lines expressing N- and C-terminally tagged variants of ATOM14 in order to investigate its topology by protease protection assays. C-terminally c-Myc-tagged ATOM14 was resistant to the protease treatment of intact mitochondria and only digested when the membranes were solubilized by detergent (Fig. 2D, left panel). N-terminally c-Myc-tagged ATOM on the other hand was protease-sensitive even in intact mitochondria (Fig. 2D right panel). Immunoblots probed with an antiserum against the soluble IMS-localized chaperone Tim9 show that the mitochondrial OM is still intact in the isolated mitochondria that were used in the experiment (Fig. 2D, bottom panels). Thus, we conclude that, like yeast Tom22 and plant Tom9, ATOM14 is an integral membrane protein with a single membrane spanning domain whose N-terminus faces the cytosol. The fact that the band for the C-terminally tagged ATOM14 co-migrated with the one in the protease-treated sample suggests that except when carrying a N-terminal c-Myc-tag the cytosolic domain of ATOM14 is essentially protected from proteinase K treatment in intact mitochondria.

### Yeast Tom22 and plant Tom9 do not complement for lack of ATOM14

In order to investigate the contributions of the different ATOM14-domains to protein function we produced an RNAi cell line allowing Tet-inducible ablation of ATOM14. However, unlike in the original cell line in which the ORF was targeted^16^, the RNAi was directed against the 3′UTR of the ATOM14 transcript. This cell line can therefore be used to test whether variants of ATOM14 are able to complement the observed growth and protein import phenotype, provided they are expressed from a different genomic context. The terms essential or non-essential, that are used below, mean that the corresponding ATOM14 variants are either required or not required for growth of procyclic *T. brucei* cells grown in SDM79 medium as described in the Material and Methods section.

**Figure 3:**
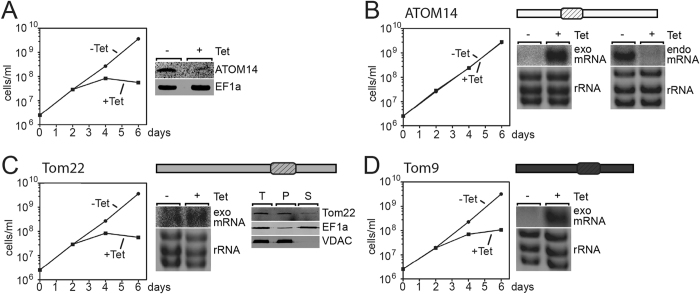
Yeast Tom22 and plant Tom9 do not complement for lack of ATOM14. (**A**) Growth curve of the uninduced (−Tet) and induced (+Tet) ATOM14 3′UTR RNAi cell line. Right panel, immunoblot probed for ATOM14 and elongation factor 1a (EF1a) which serves as a loading control. (**B**) ATOM14 3′UTR RNAi cell line ectopically expressing wildtype ATOM14 (light grey) under Tet control. The addition of Tet induces simultaneous upregulation of the ectopic copy (exo mRNA) of the ATOM14 gene and downregulation of the mRNA transcribed from the endogenous ATOM14 gene (endo mRNA), respectively. (**C**) As in (**B**) but the complementation was performed using the yeast Tom22 gene (medium grey). Right panel, immunoblot of total, pellet and supernatant fractions of a digitonin fractionation using the same complemented cell line probed with an antiserum specific for yeast Tom22. (**D**) As in (**B**) but the complementation was done using the plant Tom9 gene (dark grey). The ethidium-stained rRNAs below the Northern blots of (**B**,**C** and **D**) serve as loading controls. The position of the membrane spanning domain in the different Tom22 orthologues is indicated.

Figure 3A shows that induction of the ATOM14 3′UTR RNAi cell line results in a growth arrest. Ectopic expression of the wild-type version of ATOM14 in the same cell line as expected complements the lack of growth (Fig. 3B). The experiments in Fig. 3C and D on the other hand show that expression of yeast Tom22 or plant Tom9 fails to restore growth, suggesting that the two proteins are to diverged to take over the function of ATOM14.

For all complementation experiments Northern blots were performed to confirm that addition of Tet induces the expression of ATOM14, Tom22 and Tom9 mRNAs. Expression and localization of Tom22 was directly verified using anti-Tom22 antibodies (Fig. 3C), as no antibodies are available for Tom9 the same was not possible for Tom9.

Moreover, in experiments in which the growth phenotype was complemented Northern blots using a probe specific for the 3′UTR of the endogenous ATOM14 mRNA were performed to demonstrate Tet-inducible ablation of the endogenous ATOM14 mRNA.

### The cytosolic domain of ATOM14 is not essential

**Figure 4:**
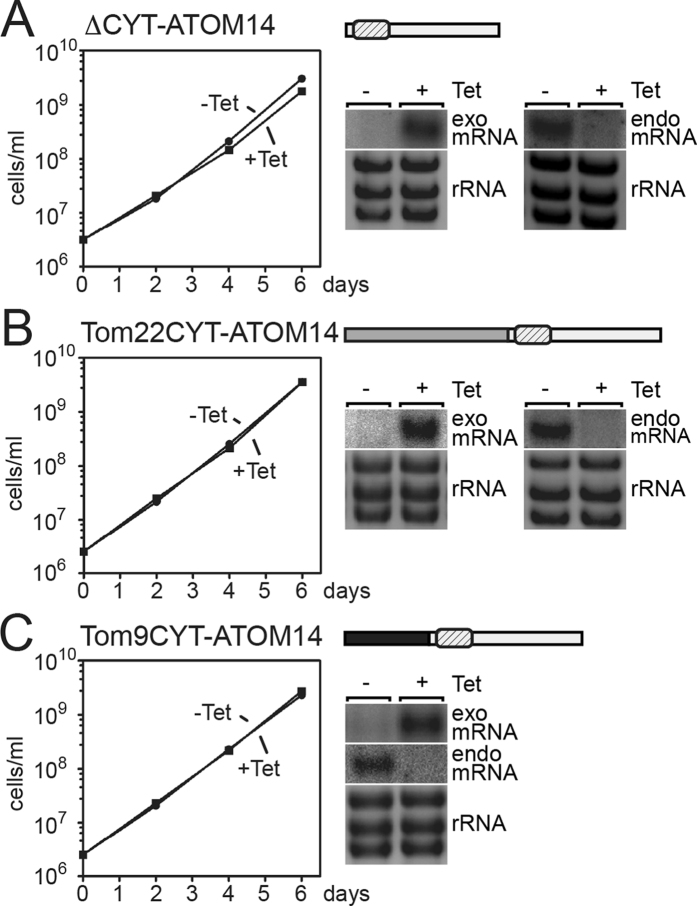
The cytosolic domain of ATOM14 is not essential. (**A**) Growth curve of an ATOM14 3′UTR RNAi cell line ectopically expressing an ATOM14 (light grey) variant lacking the cytsosolic domain. (**B**) As in (**A**) but complementation was done using an ATOM14 variant (light grey) whose cytosolic domain was replaced by the corresponding domain of yeast Tom22 (medium grey). (**C**) As in (**A** and **B**) but complementation was done using an ATOM14 variant (light grey) whose cytosolic domain was replaced by the corresponding domain of plant Tom9 (dark grey). In all three cases addition of Tet induces simultaneous upregulation of the ectopic copy (exo mRNA) of the corresponding ATOM14 variant gene and downregulation of the mRNA transcribed from the endogenous ATOM14 gene (endo mRNA), respectively. The ethidiumbromide-stained rRNAs below the Northern blots of (**A**,**B** and **C**) serve as loading controls.

ATOM14 has a short cytosolic domain that is 32 amino acids in length (Fig. 1A). To investigate its function we used the ATOM14 3′UTR RNAi cell line to express variants of ATOM14 in which essentially the whole cytosolic domain (amino acids 1 to 31) was either deleted or replaced by the corresponding domains of yeast Tom22 (amino acids 1 to 94 of Tom22) or plant Tom9 (amino acids 1 to 48 of Tom9) (Fig. 1B). In all three cases normal growth seen in uniduced cells was restored (Fig. 4, Fig. S1 in the Supplementary Information). In summary these experiments show that the cytosolic domain of ATOM14 is dispensable for protein function.

### The IMS domain of ATOM14 is essential

**Figure 5:**
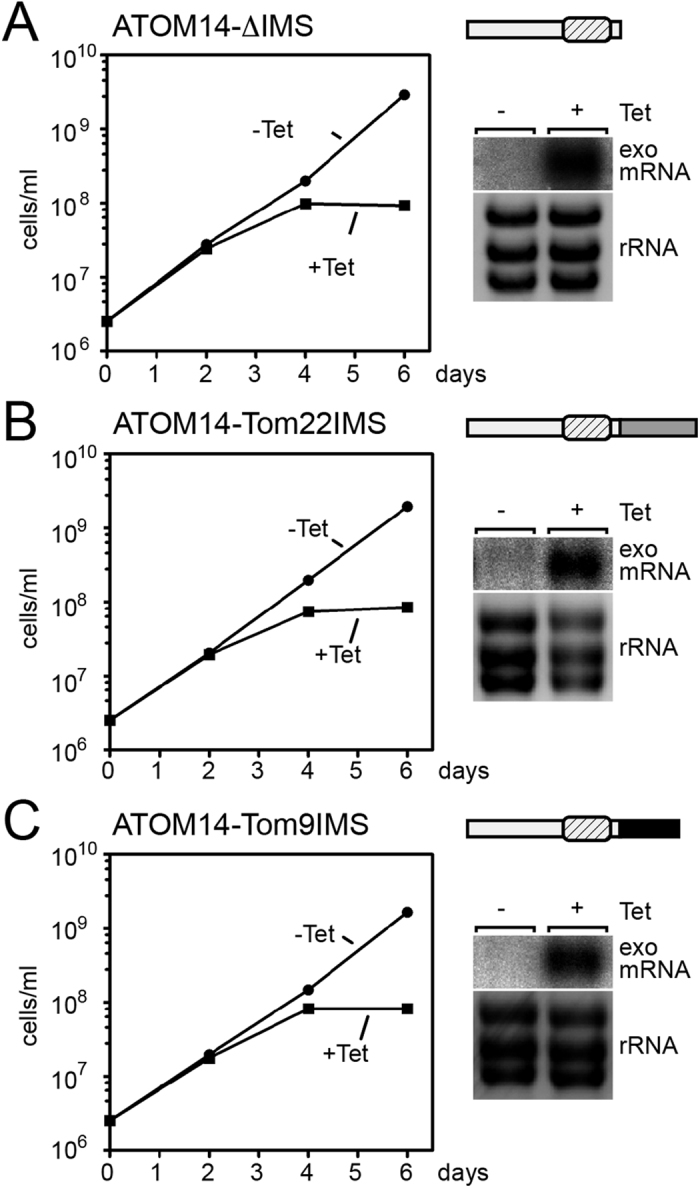
The IMS domain of ATOM14 is essential. (**A**) ATOM14 3′UTR RNAi cell line ectopically expressing an ATOM14 (light grey) variant lacking the IMS domain. (**B**) As in (**A**) but complementation was done using an ATOM14 variant (light grey) whose IMS domain was replaced by the corresponding domain of yeast Tom22 (medium grey). (**C**) As in (**A** and **B**) but complementation was done using an ATOM14 variant (light grey) whose IMS domain was replaced by the corresponding domain of plant Tom9 (dark grey). In all three cases upregulation of the ectopic copy (exo mRNA) of the corresponding ATOM14 variant gene was confirmed by Northern blots. The ethidiumbromide-stained rRNAs below the Northern blots of (**A**,**B** and **C**) serve as loading controls.

The IMS-facing domain of ATOM14 is, with 64 amino acids, significantly longer than the corresponding regions in yeast Tom22 (33 amino acids) and plant Tom9 (26 amino acids) (Fig. 1A). Figure 5 shows that a variant of ATOM14 that lacks the IMS domain is not able to complement the growth arrest observed in the ATOM14 3′UTR RNAi cell line. The same was the case for two ATOM14 variants in which the endogenous IMS domain of the protein was replaced by the corresponding shorter domains of yeast Tom22 and plant Tom9, respectively. Thus, unlike the cytosolic domain, the IMS domain is essential for ATOM14 function.

### The observed growth arrests correlate with accumulation of precursor proteins

ATOM14 is a core subunit of the ATOM complex. The reason for the observed growth arrest caused by the absence of functional ATOM14 is expected to be a reduction of mitochondrial protein import. As previously shown inhibition of import results, at least for some proteins such as cytochrome oxidase subunit IV (CoxIV) and mitochondrial heat shock protein 70 (mtHsp70), in the cytosolic accumulation of unprocessed precursor proteins which can be monitored by immunoblotting^16^,^17^. Thus, we selected the ATOM14 3′UTR RNAi cell line and three of its derivatives that were complemented with wild-type ATOM14 or its variants lacking either the IMS or the cytosolic domains, respectively, for *in vivo* analysis of mitochondrial protein import.

**Figure 6:**
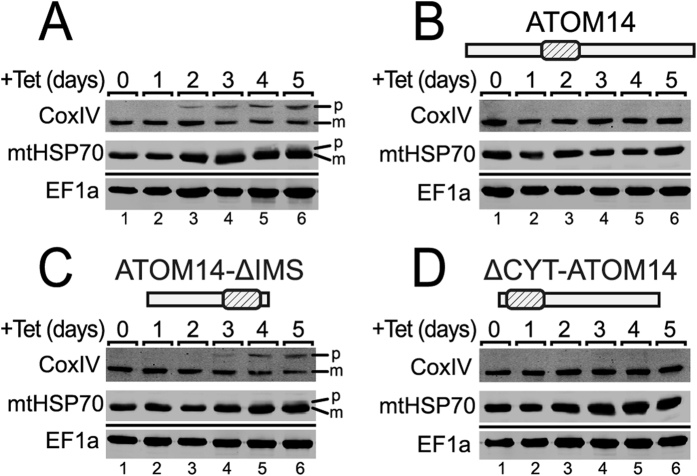
The observed growth arrests correlate with accumulation of precursor proteins. Immunoblots showing the steady-state levels of CoxIV and mtHsp70 in the uncomplemented and complemented ATOM14 3′UTR RNAi cell lines. Cytosolic EF1a serves as a loading control. Time of induction in days is indicated at the top. The position of precursor (p) and mature forms (m) are indicated. The following cell lines were used: (**A**) ATOM14 3′UTR RNAi cell line, (**B**) ATOM14 3′UTR RNAi cell line ectopically expressing wildtype ATOM14, (**C**) ATOM14 3′UTR RNAi cell line ectopically expressing an ATOM14 variant lacking the IMS domain and (**D**) ATOM14 3′UTR RNAi cell line ectopically expressing an ATOM14 variant lacking the cytsosolic domain.

The immunoblots in Fig. 6A and C show that for the two constructs that were unable to complement the growth arrest we observed an accumulation of precursor proteins. This strongly suggests that the lack of functional ATOM14 directly abolishes mitochondrial protein import.

### The IMS domain of ATOM14 interacts with precursor proteins

**Figure 7:**
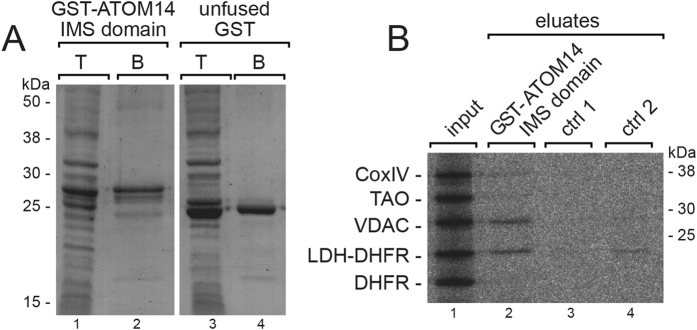
The IMS domain of ATOM14 interacts with precursor proteins. (**A**) Coomassie-stain of an SDS-gel showing total cellular extracts (T) and purified protein fractions (B) of *E. coli* cell lines expressing either the GST-tagged IMS domain of ATOM14 or unfused GST. (**B**) Binding of a mixture of precursor proteins to the Sepharose bound IMS domain of ATOM14. The gel shows an autoradiography of 2.5% of the input fraction consisting of the indicated [^35^S]-Met-labeled precursor proteins, and 100% of the eluates from the glutathione Sepharose beads. Two conditions were tested: in the first one the equal protein amounts (unfused and fused GST, ctrl 1) and in the second one equal bed volumes of the resin were compared (ctrl 2).

In order to determine the function of the IMS domain of ATOM14 in mitochondrial protein import, a chimeric protein consisting of GST fused to the IMS domain of ATOM14 was recombinantly expressed in *E. coli* and purified by glutathione affinity chromatography (Fig. 7A). Subsequently, a mixture of [^35^S]-labelled *in vitro* translated mitochondrial precursor proteins was incubated with the resin-bound GST-ATOM14 IMS domain fusion protein. After washing, bound proteins were eluted and analysed by SDS–PAGE and autoradiography (Fig. 7B). To control for non-specific binding the same experiments were performed using a glutathione resin to which unfused GST was bound. Two conditions were tested: in the first one the equal protein amounts (unfused and fused GST) (Fig. 7B, ctrl 1) and in the second one equal bed volumes of the resin were compared (Fig. 7B, ctrl 2). The results show that in line with its proposed receptor function the IMS domain of ATOM14 binds a subset of mitochondrial preproteins (Fig. 7B). VDAC and a fusion protein consisting of the 14 amino acid long N-terminal presequence of trypanosomal lipoamid dehydrogenase (LDH) that was fused to mouse dihydrofolate reductase (DHFR) bound most efficiently. Less efficient binding was also observed for the precursor of CoxIV. In contrast, neither alternative oxidase (TAO) nor unfused DHFR were recovered in the eluate. This shows that the LDH-DHFR fusion protein binding was mediated by the 14 amino acid presquence.

However, it should be considered that the assays presented in Fig. 7 are *in vitro* experiments and that the *in vivo* interactions between precursor proteins and ATOM14 might be influenced by other ATOM subunits or other proteins that are not present in the assay.

### The membrane domain of ATOM14 mediates formation of the ATOM complex

**Figure 8:**
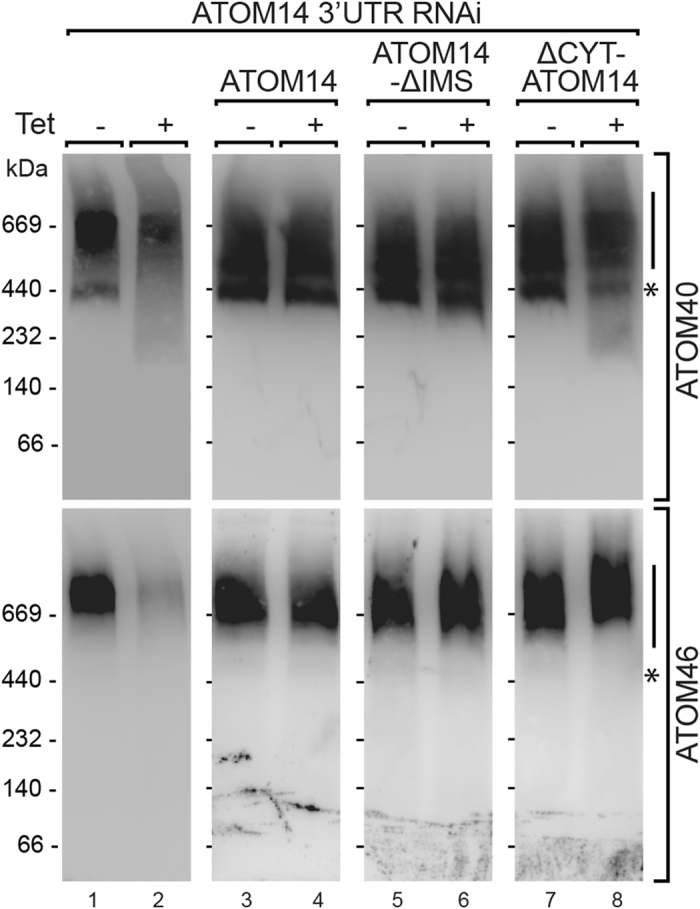
The membrane domain of ATOM14 mediates assembly of the ATOM complex. Digitonin-extracted crude mitochondrial fractions of the indicated uninduced (−Tet) and induced (+Tet) transgenic *T. brucei* cell lines were separated by BN-PAGE. The resulting immunoblots were probed using antisera against ATOM40 (top panels) and ATOM46 (bottom panels), respectively. The following cell lines were used: ATOM14 3′UTR RNAi cell line, ATOM14 3′UTR RNAi cell line ectopically expressing wildtype ATOM14 (ATOM14), ATOM14 3′UTR RNAi cell line ectopically expressing an ATOM14 variant lacking the IMS domain (ATOM14-ΔIMS) and ATOM14 3′UTR RNAi cell line ectopically expressing an ATOM14 variant lacking the cytsosolic domain (ΔCYT-ATOM14). Vertical lines on the right side of the panels indicate the position of the high molecular weight ATOM complexes. Asterisks on the right side of the panels indicate the position of the ATOM core complex.

Yeast Tom22 not only functions as a secondary receptor that binds precursor proteins on both its cytosolic and IMS domains, but also acts as an organizer of the whole TOM complex. ATOM40, the pore-forming subunit of the ATOM protein translocase^20^, occurs in up to four different protein complexes whose molecular masses range from 450 to 1000 kDa. However, only the smallest complex of 450 kDa, termed the ATOM core complex, which lacks the receptors ATOM46 and ATOM69, is essential^16^. It has been shown that ablation of ATOM14 results in a decrease of the amounts of all four ATOM complexes, including the core complex, suggesting that trypanosomal ATOM14 as Tom22 in yeast might be required for (A)TOM complex assembly^16^. In the present study we were investigating the role of the three domains of ATOM14 in the assembly of the ATOM complex. To that end, we prepared a mitochondria-enriched fraction of the ATOM14 3′UTR RNAi cell line as well as of its three derivatives complemented with ATOM14, ΔIMS-ATOM14 and ΔCYT-ATOM14. Uninduced and induced RNAi cells were solubilized by digitonin and the resulting pellets analyzed on blue native gels, which after transfer to a polyvinylidene fluoride membrane were probed with antisera recognizing the protein import channel ATOM40, which is present in all ATOM complexes, and the import receptor ATOM46, which is most abundantly found in the second largest complex, which contains less of ATOM40, and which shows the most drastic effect of all ATOM subunits upon ablation of ATOM14^16^. The results show that in the induced ATOM14 3′UTR RNAi cell line the essential ATOM core complex of 450 kDa is virtually gone and a smear corresponding to heterogenous smaller complexes becomes visible. Moreover, the second largest complex that contains the highest amounts of ATOM46 is also much reduced (Fig. 8, left upper and lower panels). As expected all these phenotypes are reverted when the cell line is complemented with the wild-type version of ATOM14. However, in the two remaining complemented cell lines the ATOM complexes, including the ATOM core complex, remained essentially intact. The cell line complemented with the ΔIMS-ATOM14 variant shows both a growth arrest (Fig. 5A) and a protein import phenotype (Fig. 6C). Thus, even though ATOM assembly is not affected in this cell line, protein import is nevertheless abolished, suggesting that binding of precursor proteins by the IMS domain of ATOM14 is essential for protein import. The ΔCYT-ATOM14 variant, on the other hand, remains functional for protein import. In this case the signal corresponding to the ATOM core complex is still clearly visible in the samples from the induced cells, although the ATOM complex architecture is somewhat disturbed as evidenced by the smear of smaller complexes. It appears that in this cell line the ATOM46-containing complexes are shifted to a slightly higher molecular weight. This has previously been observed in cells lacking ATOM12^16^. Unfortunately, we do not have a suitable antiserum which would allow us to test whether the amounts of ATOM12 are reduced in this cell lines.

In summary, our study demonstrates that ATOM14 has a receptor as well as an ATOM assembly function, both of which are independently essential for protein import. Furthermore, the results indicate that the membrane-spanning domain of ATOM14 alone is sufficient for assembly of a functional ATOM complex.

## Discussion

With the exception of Tom40, Tom22 is the only TOM complex subunit that is conserved in all eukaryotes^2^. It has a single membrane-spanning α-helix and its N-terminus faces the cytosol. Orthologues of yeast Tom22 in other Opisthokonts, are relatively easy to find using BLAST searches. However, to detect Tom22 orthologues in the other eukaryotic supergroups is more difficult and often requires sensitive methods such as structure-based HHPred analyses^18^. This is due to the fact that the proteins are rather short and there is only low sequence similarity between the Tom22 orthologues which largely is confined to the transmembrane domain and short stretches of its flanking sequence (Fig. 1A).

In our study we have investigated ATOM14 of trypanosomatids, the most highly diverged Tom22 orthologue known. It has a very short cytosolic domain that lacks the acidic clusters of amino acids that are representative of Opisthokont Tom22s. ATOM14 is essential under all conditions tested including in a bloodstream form *T. brucei* cell line that can grow in the absence of mitochondrial DNA^16^,^21^.

Yeast Tom22 was initially also reported to be an essential protein^22^, but later it was shown that depending on the used protocols viable cell lines lacking Tom22 can be obtained. However, these cells had lost their mitochondrial DNA and grew four fold slower when compared to cells devoid of mitochondrial DNA that carried a wild-type copy of Tom22^10^.

Using complementation assays in *T. brucei* cells ablated for the endogenous ATOM14, we could show that an ATOM14 variant lacking the entire cytosolic domain can restore wild-type growth. This is in contrast to a Tom22 deletion strain of the fungal species *Neurospora crassa*, where expression of a Tom22 variant lacking the N-terminal positively charged half of the cytosolic domain was not able to support growth^9^. However, in the same system complementation with a Tom22 variant in which 15 out of the 18 negatively charged residues of the cytosolic domain had been replaced by uncharged amino acids did rescue growth, indicating that the N-terminal half of the cytosolic domain but not the cluster of acidic residues are essential for normal growth and efficient mitochondrial protein import^9^.

The IMS-facing domain of ATOM14 is approximately twice as long as those of yeast Tom22 or plant Tom9 (Fig. 1). It is essential for the function of the protein as complementation of a trypanosomal ATOM14 3′UTR RNAi cell line with a variant of ATOM14 that lacks the IMS domain could neither restore growth nor mitochondrial protein import. The same result was obtained when the IMS domain of ATOM14 was replaced by those of yeast Tom22 or plant Tom9. In yeast, however, expression of a Tom22 variant carrying the IMS domain of plant Tom9 could restore wild-type growth^13^. This illustrates that, as might be expected from the sequence comparison (Fig. 1A), yeast Tom22 is also in functional terms more closely related to plant Tom9 than to ATOM14.

Whether and under which conditions the IMS domain of yeast Tom22 is essential is controversial. Two studies reported that the domain can be deleted without affecting growth of the cells on either glucose or non-fermentable carbon sources^6^,^23^. However other studies showed that in cells that exclusively express a Tom22 variant that lacks the IMS domain growth on non-fermentable carbon sources is abolished and growth on glucose is slowed down^5^,^13^.

Despite these differences, there is a consensus that the IMS domain of the yeast protein cooperates with the primary import receptors Tom20 and Tom70 and that it can bind mitochondrial precursor proteins^23^,^24^,^25^. Moreover, it has been shown that the IMS domain when not bound to a presequence can interact with the translocase of the inner membrane 23 (TIM23) complex subunit Tim50 and thus tether the TOM complex to the TIM23 complex^25^. Using the recombinantly expressed and affinity purified IMS-domain of ATOM14 we show that also in trypanosomes the IMS domain can bind precursor proteins. However, unlike the IMS domain of yeast Tom22, which selectively bound presequence-containing precursor proteins but not the β-barrel protein VDAC^24^, the IMS domain of the trypanosomal protein interacted with both proteins. This suggests that trypanosomatid ATOM14 might be required for import of a broader range of substrates than yeast Tom22.

The membrane-spanning domain of yeast Tom22 functions as an organizer of the 400 kDa TOM complex since in its absence only the 450 kDa core complex is formed^10^. In our study we show that complementation of ATOM14-ablated trypanosomes with ATOM14 variants that either lack the cytosolic or the IMS domain restores the assembly of the different ATOM complexes. This strongly suggests that the membrane-spanning domain of ATOM14, similar to its counterpart in yeast Tom22, is necessary and sufficient for assembly of the high molecular weight translocase complexes. However, unlike for yeast Tom22 absence of ATOM14 does not result in the accumulation of a low molecular weight complex^10^ but rather results in a decline of all translocase complexes.

The cytosolic domain of Tom22 on the other hand seems less conserved. It is either long and has clusters of positively charged residues or it is shorter and lacks these features^2^. The former variants are restricted to the Opisthokonts and therefore likely evolved in this lineage. However even in this group we find at least one example, the Tom22 of *Saccharomyces castelii,* that lacks the positively charged clusters. It has been suggested in this case that this lack is compensated for by the gain of an acidic cluster in the cytosolic domain of the primary receptor Tom20^26^. All other eukaryotic supergroups appear to have Tom22 orthologues with shorter cytosolic domains that lack the clusters of positively charged amino acids^2^. In plants it has been shown that this domain may not necessarily be able to bind presequences^27^. ATOM14 is unusual in that it carries the shortest such domain known. In plants and in trypanosomes the composition of the outer membrane protein translocase is known^11^,^12^,^16^. There is no evidence that the absence of acidic clusters in Tom9 and ATOM14, as in *S. castelii*, is compensated for by a gain of such a domain in any of the receptor subunits^26^.

In summary, our study shows that IMS-exposed domains which can bind presequences and membrane-spanning domains that mediate assembly of the translocase subunits to high molecular weight complexes are functionally conserved between Tom22 in yeast and ATOM14 in trypanosomes. This strongly suggests that despite the limited structural similarities between the two proteins they are indeed orthologues that are derived from a common ancestor. Moreover, since trypanosomatids belong to the earliest diverging eukaryotes^14^,^15^ it follows that these features likely are conserved in Tom22-like proteins of all eukaryotes.

## Material and Methods

### Transgenic cell lines

All transgenic cell lines are derived from the procyclic *T. brucei* strain 29–13^28^. Cells were grown in SDM-79 medium^29^ containing 10% (v/v) foetal calf serum at 27 °C. To allow for inducible knockdown of ATOM14 (Tb927.11.5600) and simultaneous expression of truncated ATOM14 variants or fusions between ATOM14 and yeast Tom22 (YNL131W) or plant Tom9 (AT5G43970.1), respectively, a 441 bp long fragment starting from position 4 after the stop codon of the ATOM14 3′ untranslated region (3′UTR) was selected as the RNAi target region. RNAi was done using a stemloop vector based on the plasmid pLEW100 in which the phleomycine resistance gene has been replaced by a blasticidine resistance gene and which allows for ligation of RNAi fragments in opposing directions using BamHI/XhoI and HindIII/XbaI restriction sites separated by a 460 bp spacer region^28^,^30^. ATOM14 and Tom22 sequences were PCR-amplified from *T. brucei* 427 and *S. cerevisiae* genomic DNA and Tom9 sequences from *Arabidopsis thaliana* Col-0 leaf cDNA. Inducible expression of exogenous proteins was done using a modified pLEW100 vector in which the phleomycine resistance gene has been replaced by a puromycine resistance gene. Fusions of ATOM14 with the IMS domains of Tom22 or Tom9 were made by overlap extension PCR using ATOM14-specific PCR amplicons and long synthetic oligonucleotide primers corresponding to the IMS portion of Tom22 and Tom9, respectively. Fusions of ATOM14 with the cytosolic domains of Tom22 or Tom9 were made by overlap extension PCR using PCR amplicons specific for the respective ATOM14 and Tom22 or Tom9 sequences. For exact sequences of protein fusions see Fig. 1B. Triple c-Myc-tagging of ATOM14 and *in situ* HA-tagging of ATOM40 has been described elsewhere^16^.

### Digitonin extraction

Cells were induced with tetracycline (Tet) to express N-terminally and C-terminally triple c-Myc-tagged ATOM14. Cells were washed in PBS and resuspended in SoTE (20 mM Tris-HCl pH 8.0, 2 mM EDTA pH 8.0, 600 mM sorbitol) containing 0.015% (w/v) digitonin to lyse the plasma membrane. After differential centrifugation (6.800 rcf, 5 min, 4 °C) a mitochondria-enriched pellet fraction and a fraction enriched for cytosolic components were obtained^30^.

### Isolation of mitochondria

Intact mitochondria were isolated under isotonic conditions as described^31^,^32^. Briefly, cells induced with Tet to express triple c-Myc-tagged ATOM14 were washed with SBG buffer (22 mM glucose, 150 mM NaCl, 20 mM sodium phosphate buffer pH 7.9) and resuspended in SoTE. Following cell breakage by nitrogen cavitation (55 bar, 30 min) and centrifugation (23.400rcf_max_, 4 °C, 10 min), the resulting pellet was taken up in SoTE and incubated in presence of DNase I. The material was concentrated by centrifugation (23.400 rcf_max_, 4 °C, 10 min) and subsequently separated on a Nycodenz step gradient (28%, 25%, 21% and 18% in SoTE). Highly pure mitochondria were isolated from the gradient after ultracentrifugation (125.000 rcf_max_, 4 °C, 45 min).

### Carbonate extraction

Mitochondria (100 μg) isolated under isotonic conditions and containing C-terminally triple c-Myc-tagged ATOM14 were resuspended in 160 μl of 100 mM Na_2_CO_3_ pH 11.5 and incubated on ice for 10 min. 80 μl was withdrawn and mixed with 40 μl 3X SDS sample buffer to serve as ‘total’ sample. The remaining 80 μl were centrifuged (100.000 rcf, 10 min, 4 °C) and the supernatant and the pellet, after resuspension in 80 μl 100 mM Na_2_CO_3_ pH 11.5, were mixed with 40 μl 3X SDS sample buffer. Equal cell equivalents of each sample were analysed by immunoblotting.

### Immunoprecipitation

Purified mitochondria containing C- or N-terminally triple c-Myc-tagged ATOM14 or mitochondria-enriched fractions derived from digitonin extractions of cells expressing C-terminally triple c-Myc-tagged ATOM14 and *in situ* triple HA-tagged ATOM40 were used for immunoprecipitations^16^. The material was lysed on ice for 15 min in lysis buffer (20 mM Tris-HCl pH 7.4, 0.1 mM EDTA, 100 mM NaCl, 25 mM KCl, complete protease inhibitor cocktail EDTA-free (Roche)) containing 1.5% (w/v) digitonin. The lysate was cleared by centrifugation (18.000 rcf, 4 °C, 15 min). From the resulting supernatant an input sample (5%) was withdrawn and the rest was incubated for 2 h at 4 °C in presence of anti-c-Myc agarose beads (Clontech Laboratories, Inc., Product No. 631208; Sigma, Product No. E6654) or anti-HA agarose beads (Roche Applied Science, Product No. 11815016001). Beads were washed three times with lysis buffer containing 0.2% (w/v) digitonin prior to elution by boiling in SDS sample buffer.

### Protease protection assay

Isotonically isolated mitochondria (25 μg) containing C- or N-terminally triple c-Myc-tagged ATOM14 were resuspended in 20 mM Tris-HCl pH 7.2, 15 mM KH_2_PO_4_, 20 mM MgSO_4_, 0.6 M sorbitol in a volume of 50 μl with and without proteinase K (10 μg/ml) and 0.5% (v/v) Triton-X100 and incubated on ice for 15 min. To stop the reactions PMSF was added to a concentration of 5 mM and mitochondria were collected by centrifugation (6.800 rcf, 4 °C, 5 min) and subsequently resuspended in SDS sample buffer and analysed by immunoblotting.

### Northern blotting

Acid guanidinium thiocyanate-phenol-chloroform extraction^33^ was used to isolate total RNA from uninduced and induced cells. RNA was separated on a 1% (w/v) agarose gel in 20 mM MOPS-KOH buffer pH 7.0 containing 0.5% (v/v) formaldehyde. Northern probes complementary to the ATOM14 3′UTR to confirm the successful knockdown of ATOM14 and to the aldolase 3′UTR to check expression of inducible exogenous mRNAs were generated by PCR from genomic DNA of *T. brucei* 427, gel-purified and radioactively labelled using the Prime-a-Gene labelling system (Promega).

### Binding assay

The C-terminal IMS domain of ATOM14 (amino acids 59–119) was fused to an N-terminal GST tag using the pGEX-6P-1 vector and expressed in *Escherichia coli*. The GST-tagged fusion protein and unfused GST were purified using glutathione sepharose 4B (GE Healthcare). Briefly, bacterial cultures were grown in LB medium containing 100 μg/ml ampicillin to an OD_600_ of 0.6 at 37 °C followed by induction with 1 mM isopropyl-β-D-thiogalactopyranosid for 3 h. 100 ml of culture were harvested and pellets resuspended in 10 ml lysis buffer (20 mM Tris-HCl pH 7.6, 300 mM NaCl, 1 mM EDTA, complete protease inhibitor EDTA-free (Roche)). Cells were lysed by sonication on ice followed by centrifugation (26.000 g, 4 °C, 30 min). The supernatant was subsequently incubated with 100 μl of glutathione sepharose on a rotator (1 h, 4 °C) followed by washing of the beads with lysis buffer. The protein-loaded beads were stored in lysis buffer at 4 °C for a maximum of 24 h prior to use. Purity and protein amounts were determined by SDS-PAGE and comparison to a bovine serum albumin standard. [^35^S]-Met-labelled precursor proteins LDH (amino acids 1–14)-DHFR, VDAC (Tb927.2.2510), CoxIV (Tb927.1.4100) and TAO (Tb927.10.7090) were synthesized *in vitro* using the TNT T7 Quick for PCR (Promega) or the TNT SP6 Quick for PCR (Promega) coupled transcription/translation systems as described before^16^. Radioactive precursor proteins were quantified by SDS-PAGE and autoradiography and mixed accordingly in order to yield equal signal intensity. For binding assays, a bead volume containing approximately 400 pmol of the GST-tagged ATOM14 IMS domain fusion protein was used. An equal amount of unfused GST or an equal volume of beads loaded with unfused GST served as negative controls. The beads were washed three times with 500 μl of assay buffer (10 mM MOPS-KOH pH 7.2, 400 mM KCl, 1% BSA (w/v), 0.5% digitonin (w/v)) and then resuspended in 150 μl assay buffer. To each binding reaction 16.5 μl of the precursor mix was added followed by incubation at 27 °C for 1 h. Beads were washed three times with 500 μl assay buffer and eluted with 300 μl of elution buffer (20 mM Tris-HCl pH7.6, 300 mM NaCl, 1 mM EDTA, 20 mM glutathione). The eluted proteins were precipitated with TCA and analysed on a 13% SDS-PAGE along with an input sample (2.5%).

### Blue-native PAGE

Mitochondria-enriched fractions derived by digitonin extraction were solubilized in a buffer (20 mM Tris-HCl pH 7.4, 50 mM NaCl, 10% glycerol and 0.1 mM EDTA) containing 1.5% (w/v) digitonin. Extracts were cleared by centrifugation prior to separation on 4–13% gradient gels. Gels were soaked in SDS-PAGE running buffer (25 mM Tris, 1 mM EDTA, 190 mM glycine, 0.05% (w/v) SDS) in order to facilitate the transfer of proteins to membranes and subsequent immunoblotting.

### Antibodies

The following antibodies were used in this study: rabbit anti-ATOM14 (dilution 1:500), rabbit anti-ATOM69 (dilution 1:50), rabbit anti-ATOM46 (dilution 1:50), rabbit anti-ATOM11 (dilution 1:50)^16^, rabbit anti-ATOM40 (dilution 1:1000), rabbit anti-VDAC (dilution1:1000), rabbit anti-CoxIV (dilution 1:1000), rabbit anti-cytochrome C (Cyt C) (dilution 1:100), rabbit anti-Tim9 (dilution1:20)^34^. Mouse anti-c-Myc (Invitrogen, Product No. 132500, dilution 1:2000), mouse anti-HA (Enzo Life Sciences AG, Product No. CO-MMS-R-1000, dilution 1:5000) mouse anti-elongation factor 1a (EF1a) (Merck Millipore, Product No. 05–235, dilution 1:10.000). Rabbit anti-mitochondrial heat shock protein 70 (mtHsp70) (dilution 1:1000) was kindly provided by R. Jensen.

## Additional Information

**How to cite this article:** Mani, J. *et al*. Mitochondrial protein import - Functional analysis of the highly diverged Tom22 orthologue of *Trypanosoma brucei. Sci. Rep.* **7**, 40738; doi: 10.1038/srep40738 (2017).

**Publisher's note:** Springer Nature remains neutral with regard to jurisdictional claims in published maps and institutional affiliations.

## Supplementary Material

Supplementary Fig. S1
