# Supplementary material for: Mitochondrial protein import - Functional analysis of the highly diverged Tom22 orthologue of Trypanosoma brucei
Source: Sci Rep. 2017 Jan 17;7:40738. doi: 10.1038/srep40738 (PMC5240110; doi:10.1038/srep40738)
Supplement: Supplementary Fig. S1 [file srep40738-s1.pdf]

Supplementary Information

Mitochondrial protein import - Functional analysis of the highly diverged Tom22 orthologue of *Trypanosoma brucei*

Jan Mani, Sam Rout, Silvia Desy, and André Schneider
From the Department of Chemistry and Biochemistry, University of Bern, Freiestrasse 3, CH-3012 Bern, Switzerland
To whom correspondence should be addressed: André Schneider, Department of Chemistry and Biochemistry, University of Bern, Freiestrasse 3, CH-3012 Bern, Switzerland, Telephone: +41 (0)31 631 4253; E-mail: andre.schneider@dcb.unibe.ch

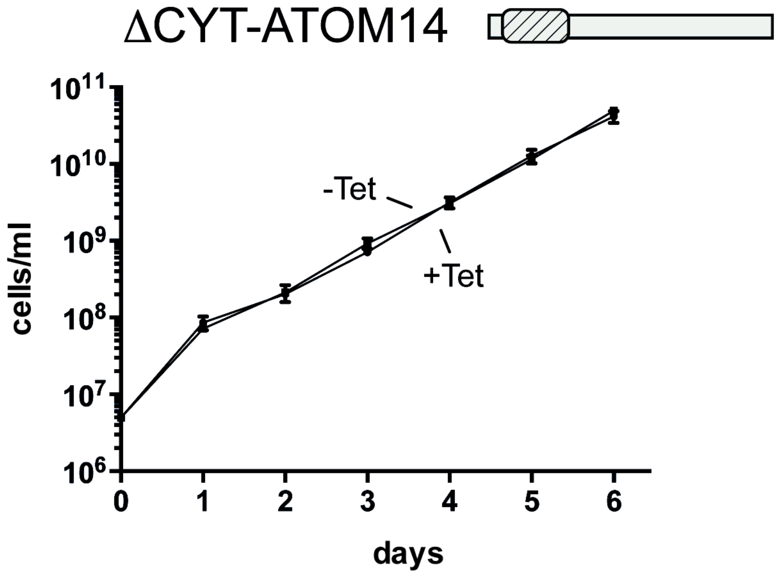

SUPPLEMENTARY FIGURE S1. **The cytosolic domain of ATOM14 is not essential for normal growth.** Triplicate growth curves of the ATOM14 3'UTR RNAi cell line ectopically expressing an ATOM14 (light grey) variant lacking the cytosolic domain are shown. Standard errors are indicated.
